# Familial or Sporadic Idiopathic Scoliosis – classification based on artificial neural network and GAPDH and ACTB transcription profile

**DOI:** 10.1186/1475-925X-12-1

**Published:** 2013-01-04

**Authors:** Tomasz Waller, Roman Nowak, Magdalena Tkacz, Damian Zapart, Urszula Mazurek

**Affiliations:** 1 Institute of Computer Science, Division of Biomedical Computer Systems, University of Silesia, Katowice, Poland; 2 Department and Clinic of Orthopaedics Medical University of Silesia, Sosnowiec, Poland; 3 Department of Molecular Biology, Medical University of Silesia, Katowice, Poland; 4 Institute of Computer Science, Division of Information Systems, University of Silesia, Katowice, Poland

## Abstract

**Background:**

Importance of hereditary factors in the etiology of Idiopathic Scoliosis is widely accepted. In clinical practice some of the IS patients present with positive familial history of the deformity and some do not. Traditionally about 90% of patients have been considered as sporadic cases without familial recurrence. However the exact proportion of Familial and Sporadic Idiopathic Scoliosis is still unknown. Housekeeping genes encode proteins that are usually essential for the maintenance of basic cellular functions. ACTB and GAPDH are two housekeeping genes encoding respectively a cytoskeletal protein β-actin, and glyceraldehyde-3-phosphate dehydrogenase, an enzyme of glycolysis. Although their expression levels can fluctuate between different tissues and persons, human housekeeping genes seem to exhibit a preserved tissue-wide expression ranking order. It was hypothesized that expression ranking order of two representative housekeeping genes ACTB and GAPDH might be disturbed in the tissues of patients with Familial Idiopathic Scoliosis (with positive family history of idiopathic scoliosis) opposed to the patients with no family members affected (Sporadic Idiopathic Scoliosis). An artificial neural network (ANN) was developed that could serve to differentiate between familial and sporadic cases of idiopathic scoliosis based on the expression levels of ACTB and GAPDH in different tissues of scoliotic patients. The aim of the study was to investigate whether the expression levels of ACTB and GAPDH in different tissues of idiopathic scoliosis patients could be used as a source of data for specially developed artificial neural network in order to predict the positive family history of index patient.

**Results:**

The comparison of developed models showed, that the most satisfactory classification accuracy was achieved for ANN model with 18 nodes in the first hidden layer and 16 nodes in the second hidden layer. The classification accuracy for positive Idiopathic Scoliosis anamnesis only with the expression measurements of ACTB and GAPDH with the use of ANN based on 6-18-16-1 architecture was 8 of 9 (88%). Only in one case the prediction was ambiguous.

**Conclusions:**

Specially designed artificial neural network model proved possible association between expression level of ACTB, GAPDH and positive familial history of Idiopathic Scoliosis.

## Background

Idiopathic Scoliosis (IS) is the most common structural deformity of the human spine. Although the exact cause or causes of idiopathic scoliosis are still unknown there is convincing evidence supporting a genetic aetiology of this disorder [1-5]. Importance of hereditary factors in the etiology of IS is underlined by increased risk of scoliosis among the first-degree relatives of index patients. Harrington found scoliosis incidence of 27% among the first degree relatives. [6] Other studies indicate 11% of first degree and 2,4% and 1,4% of second and third degree relatives are affected [7,8]. Genetic basis of IS is also supported by the results of the twin studies. Inoue and colleagues found the concordance rate of scoliosis of 92,3% in monozygous, decreasing to 62,5% in dizygous twins [9]. Lower concordance rate was found in the study of Kesling and al, 73% among monozygous and 36% among dizygous twins [10]. Recent study based on the Swedish Twin Registry estimates that heritability of this condition is 38% indicating the importance of other still unknown factors in the development of the deformity [11]. Mode of inheritance and genetic basis of the scoliotic phenotype are still not definitively determined. Autosomal dominant mode, X-linked as well as multifactorial inheritance patterns were suggested [3-7]. According to Miller et al. idiopathic scoliosis is a complex genetic disorder involving one or more genetic loci and complex genetic interactions for disease expression [5]. In clinical practice some of the IS patients present with positive familial history of the deformity and some do not. Traditionally about 90% of patients have been considered as sporadic cases without familial recurrence [1]. However the exact proportion of Familial and Sporadic Idiopathic Scoliosis is still unknown [5]. Ogilvie et al. in the population study based on a large data base from Utah conclude that 97% of their patients with idiopathic scoliosis have familial origins and suggest a presence of one or more major gene defects or single gene defects influenced by other factors [11]. According to Cheng et al. predisposition for IS doesn’t have a specific assigned risk of heritability, but inheritance is based on multiple factors potentially both genetic and environmental, which have yet to be defined [1].

Housekeeping genes encode proteins that are usually essential for the maintenance of basic cellular functions. They are expressed constitutively in every human cell but it appears that their transcriptional level may be influenced by numerous factors [12,13]. ACTB and GAPDH are two housekeeping genes encoding respectively a cytoskeletal protein β-actin, and glyceraldehyde-3-phosphate dehydrogenase, an enzyme of glycolysis [12]. Based on the assumption of their constant expression in various tissues these genes serve as traditional internal control in a variety of assays in molecular biology [13]. Although their expression levels can fluctuate between different tissues and persons, human housekeeping genes seem to exhibit a preserved tissue-wide expression ranking order [14].

It was hypothesized that expression ranking order of two representative housekeeping genes ACTB and GAPDH might be disturbed in the tissues of patients with Familial Idiopathic Scoliosis (with positive family history of idiopathic scoliosis) opposed to the patients with no family members affected (Sporadic Idiopathic Scoliosis). In order to recognize potentially sophisticated patterns in the data and because of the tensor structure of the ACTB and GAPDH expression an artificial neural network (ANN) was developed that could serve to differentiate between familial and sporadic cases of idiopathic scoliosis based on the expression levels of ACTB and GAPDH in different tissues of scoliotic patients.

The aim of the study was to investigate whether the expression levels of ACTB and GAPDH in different tissues of idiopathic scoliosis patients could be used as source of data for specially developed artificial neural network in order to predict the positive family history of index patients.

## Methods

### Patients

Study design was approved by Bioethical Committee Board of Silesian Medical University. Informed, written consent was obtained from each patient participating in the study and if required from their parents. Twenty nine patients (23 females and 6 males) with a definite diagnosis of late onset Idiopathic Scoliosis were included in the study. Thirteen of them (44%) had positive familial history of IS. All of the patients had undergone posterior corrective surgery with segmental spinal instrumentation according to C-D method. The mean age at surgery was 16 years 8 months (range 13,7 – 24 years). Based on Lenke classification 6 curves were of type 1,6 curves of type 2,7 curves of type 3,7 curves of type 4,4 curves of type 5 and 3 of type 6 [15]. Preoperatively the average frontal and sagittal Cobb angles measured on standard p-a and lateral radiograms were 68,8° (range 36°-114°) and 35,4° (range 12°-70°) respectively. The axial plane deformity was measured on CT scans performed at the curve apex by spinal rotation angle relative to sagittal plane RAsag and rib hump index RHi as described by Aaro and Dahlborn [16]. The mean RAsag was 19,3° (range 2,5°-46°) and RHi 0,4 (range 0,03-0,91). During surgery bilateral facet removal was performed in the routine manner and bone specimens from inferior articular spinal processes at the curve apex concavity and convexity were harvested. In the same time bilateral samples of paravertebral muscle tissue at the apical level and 10 ml of patients peripheral blood were collected. Every sample of bone and muscular tissue as well as blood specimens were placed in separate sterile tubes, adequately identified and immediately snap frozen in liquid nitrogen and stored at -80°C until molecular analysis.

### Laboratory procedures

Tissues samples were homogenized with the use of Polytron® (Kinematyka AG). Total RNA was isolated from tissue samples with the use of TRIZOL® reagent (Invitrogen Life Technologies, California, USA), according to the manufacturer’s instructions. Extracts of total RNA were treated with DNAase I (Qiagen Gmbh, Hilden, Germany) and purified with the use of RNeasy Mini Spin Kolumn (Qiagen Gmbh, Hilden, Germany), in accordance with manufacturer’s protocol. The quality of RNA was estimated by electrophoresis on a 1% agarose gel stained with ethidium bromide. The RNA concentration was determined by absorbance at 260 nm using a Gene Quant II spectrophotometer (Pharmacia LKB Biochrom Ltd., Cambridge, UK). Total RNA served as a matrix for QRT PCR.

ACTB and GAPDH mRNA quantification in osseous, muscular and blood tissue samples by Quantitative Real Time Reverse Transcription Polymerase Chain Reaction.

The quantitative analysis was carried out with the use of Sequence Detector ABI PRISM™ 7000 (Applied Biosystems, California, USA). The standard curve was appointed for standards of ACTB (TaqMan® DNA Template Reagents Kit, Applied Biosystems, Foster, CA, USA). The ACTB and GAPDH mRNA abundance in all studied tissue specimens were expressed as mRNA copy number per 1 μg of total RNA.

The QRT-PCR reaction mixture of a total volume of 25 μl contained QuantiTect SYBR- Green RT-PCR bufor containing Tris–HCl (NH_4_)_2_SO_4_, 5 mM MgCl_2_, pH 8,7, dNTP mix fluorescent dye SYBR-Green I, and passive reference dye ROX mixed with 0,5 μl QuantiTect RT mix (Omniscript reverse transcriptase, Sensiscript reverse transcriptase) (QuantiTect SYBR-Green RT-PCR kit; Qiagen) forward and reverse primers each at a final concentration of 0,5 μM mRNA and total RNA 0,25 μg per reaction. Sequence for primers: mRNA for mRNA for ACTB 5’TCACCCACACTGTGCCC ATCTACGA3’(forward primer) 5’CAGCGGAACCGCTCATTGCCAATGG3’ (reverse primer), mRNA for GAPDH 5’GAAGGTGAAGGTCGGAGTC3’(forward primer) 5’GAAGATGG TGATGGGATT 3’(reverse primer). Reverse transcription was carried out at 50°C for 30 min. After activation of the HotStar Taq DNA polymerase and deactivation of reverse transcriptases at 95°C for 15 min, subsequent PCR amplification consisted of denaturation at 94°C for 15 sec, annealing at 60°C for 30 sec and extension at 72°C for 30 sec (40 cycles). Final extension was carried out at 72°C for 10 min. QRT-PCR specificity was assessed by electrophoresis in 6% polyacrylamid gel and melting curves for aplimeres.

### Patient data

The results of laboratory procedures and the family anamnesis of 29 patients were used to create dataset consisting of 29 rows. The expression values were transformed to logarithmic scale. One row represented ACTB and GAPDH transcription profile in three kinds of tissue (bone, muscle, and blood) for exactly one patient.

Unfortunately, there were some missing data in our dataset. To face this problem we could either remove incomplete records from the analyzed dataset or use appropriate methodology and tool to preserve and utilize them in the analysis. In data mining and knowledge discovery from data disciplines the problem of missing data is widely discussed [17-22]. With the removal of all incomplete records we could risk losing some important information contained in the whole dataset. In effect we decided to preserve all the records and replace missing values by random data from normal distributions similar to the original distributions of the variables. The random values were marked in bold [Tables 1 and 2]. Our decision was supported by the experience of one of the co-authors of this study conducting extensive research in the field of advanced data processing therein in processing incomplete data [23-27]. Basing on the mentioned above ANN was chosen as an appropriate method for classification in this case. The dataset was randomly divided into training set (20 rows) [Table 1] and test set (9 rows) [Table 2].

**Table 1:** Training set

**ID**	**GAPDH bone** (**concavity**)	**ACTB bone** (**concavity**)	**GAPDH muscle** (**concavity**)	**ACTB muscle** (**concavity**)	**GAPDH blood**	**ACTB blood**	**CLASSIFICATION**
							**0** – **Sporadic IS**
							**1** – **Familial IS**
1	0	3,655234507	5,19447549	5,492043421	**0**,**788036615**	**7**,**947055432**	1
2	2,260071388	2,681241237	5,492043421	5,19447549	4,351834943	4,379378045	0
3	1,361727836	0	**6**,**883054459**	**1**,**932946816**	3,543198586	7,878946654	0
4	2,26245109	4,490393961	5,010236335	5,166876908	4,484314078	4,062506775	1
5	0,77815125	4,52146499	**5**,**239966296**	**4**,**435937313**	1,838849091	5,133344071	1
6	0	4,15192118	5,160648574	4,706444663	2,46686762	4,210666244	1
7	0	1,342422681	2,822821645	3,140193679	2,041392685	3,729488769	0
8	0	1,740362689	**8**,**416773187**	**1**,**307737902**	1,886490725	3,820595497	0
9	2,7084209	3,68797462	**7**,**906948855**	**4**,**842939908**	2,283301229	3,062205809	1
10	1,77815125	3,496376054	2,559906625	3,126131407	4,614992076	4,872779577	0
11	0	6,053585081	0	4,704202011	**5**,**753376838**	**0**,**8874258**	0
12	0	3,331022171	4,2175629	4,527707216	5,322554193	5,961483267	0
13	1,204119983	4,060168812	3,882068944	4,669075022	5,979840083	4,431492425	0
14	10,59979533	0	3,257198426	4,146065989	3,834102656	3,931152639	1
15	0	1,146128036	0,903089987	3,761401557	2,380211242	5,055026472	1
16	0	2,765668555	0	3,709015417	3,087426457	4,830563008	0
17	0	1,792391689	0	4,713734083	3,098643726	5,036968055	0
18	4,443841661	2,615950052	1,505149978	4,414388327	3,128076013	4,702835345	1
19	7,280817804	2,555094449	0	2,029383778	0	2,271841607	0
20	0	0,301029996	1,944482672	4,584489532	3,036628895	4,451325808	1

The random values are marked in bold.

**Table 2:** Test set and ANN’s prediction

**ID**	**GAPDH bone** (**concavity**)	**ACTB bone** (**concavity**)	**GAPDH muscle e** (**concavity**)	**ACTB muscle** (**concavity**)	**GAPDH blood**	**ACTB blood**	**CLASSIFICATION**	**6** - **18**–**16** - **1**	**6** - **19**–**19** - **1**	**6** - **18**–**10** - **1**
							**0** – **Sporadic IS**	**ANN**’**S PREDICTION**	**ANN**’**S PREDICTION**	**ANN**’**S PREDICTION**
							**1** – **Familial IS**			
21	0	2,999130541	2,086359831	4,54961624	2,595496222	5,315582034	1	0,8150237	0,0658464	0,9729949
22	0	3,325104983	2,981365509	3,940018155	3,253822439	5,188225173	0	0,1371327	0,0111759	0,9755903
23	4,259641653	4,832872801	**6**,**266957346**	**4**,**19709909**	4,228759555	4,237141427	1	0,9970080	0,8874323	0,9444540
24	1,041392685	3,29136885	6,118608586	5,357498429	**1**,**036299441**	**3**,**74587204**	1	0,9984768	0,9984370	0,9981375
25	0,84509804	4,850768727	1,176091259	3,542451947	2,305351369	3,743744879	0	0,0203165	0,0046074	0,0195937
26	2,598790507	4,308116016	5,95395578	5,604965452	3,836324116	3,596047008	1	0,9979908	0,9941236	0,9964907
27	0	3,804275767	**5**,**556972498**	**3**,**33701319**	3,055760465	3,388811413	0	0,3232977	0,2974088	0,0672966
28	0	3,513483957	**0**,**805059074**	**2**,**195759967**	1,176091259	3,688508808	0	0,0371065	0,0023705	0,0179424
29	0	4,132643851	0,84509804	4,909245708	1,579783597	4,111497749	0	0,0338294	0,0341857	0,0104404

The random values are marked in bold.

### Artificial neural network

Artificial neural network is a mathematical model that is inspired by the structure and functional aspects of biological neural networks [28,29]. ANN can be used to detect sophisticated patterns in data. Several studies have applied neural networks in research and analysis of various diseases (i.e. classification of cardiovascular disease, forecast for bacteria – antibiotic interactions, prediction of colorectal cancer patient survival) [30].

**Figure 1:**
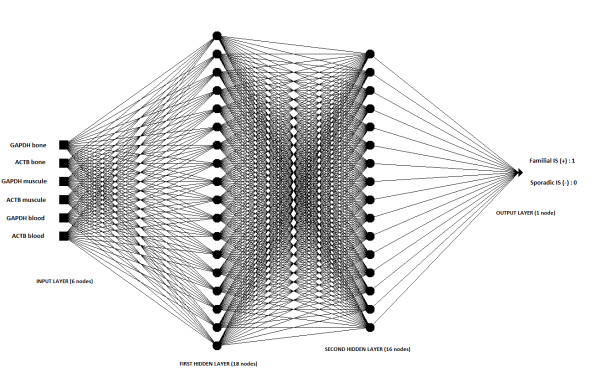
**A schematic representation of one of tested artificial neural networks.** Our ANN has input layer, two hidden layers and an output layer. The input layer has 6 neurons, the first hidden layer has 18 neurons, the second hidden layer has 16 neurons and output layer has 1 neuron.

The architecture of the ANN used in this study is the multilayered feed-forward network architecture with four layers (two hidden layers). Multilayer feed-forward neural networks can be used to approximate a nonlinear functions which are applied to describe the complicated relationships in biological data [31]. The schematic representation of the best architecture of artificial neural network for our problem is shown in Figure 1. The number of neurons in the input layer was 6 and it was equal to the number of ACTB and GAPDH expression measurements. The ideal outputs were set at 1 for the positive history of IS in the family and at 0 for absence of IS in the anamnesis. The number of hidden nodes was obtained by trial and error method. We trained 421 neural networks models with different number of hidden nodes using the backpropagation algorithm (activation function: binary sigmoidal function, learning rate: 0,1; momentum rate: 0,01; epochs: 50, 500 and 5000) and the training set. The backpropagation teaching method was chosen because it is the most common method of training multilayered feed-forward neural networks [30]. Initially, 50 training epochs were considered but it did not yield a satisfactory result (Table 3). The mean square error (MSE) was high. This MSE was minimized by increasing the epochs from 50 to 500 and finally from 500 to 5000 [Table 3]. Thereafter, we selected 3 neural networks with the least mean square error (MSE) for training set. To test the classification ability of the ANN approach, we used the selected neural models and test set of data. The ANN model with the best classification accuracy for Idiopathic Scoliosis in the anamnesis with expression measurement of ACTB and GAPDH was chosen as the best.

**Table 3:** Evaluation and selection of multiple network architectures

**No.**	**ANN architecture**	**MSE 50 epochs**	**MSE 500 epochs**	**MSE 5000 epochs**	**No.**	**ANN architecture**	**MSE 50 epochs**	**MSE 500 epochs**	**MSE 5000 epochs**	**No.**	**ANN architecture**	**MSE 50 epochs**	**MSE 500 epochs**	**MSE 5000 epochs**	**No.**	**ANN architecture**	**MSE 50 epochs**	**MSE 500 epochs**	**MSE 5000 epochs**
**1**	**6 - 18–16 - 1**	**0**,**426**	**0**,**058**	**0**,**006**	57	6 - 16–8 - 1	0,355	0,048	0,008	113	6 - 12–7 - 1	0,432	0,059	0,009	169	6 - 5–11 - 1	0,490	0,308	0,011
**2**	**6** - **19**–**19** - **1**	**0**,**413**	**0**,**049**	**0**,**006**	58	6 - 16–11 - 1	0,328	0,046	0,008	114	6 - 11–1 - 1	0,384	0,069	0,009	170	6 - 8–9 - 1	0,483	0,057	0,011
**3**	**6** - **18**–**10** - **1**	**0**,**440**	**0**,**045**	**0**,**006**	59	6 - 19–15 - 1	0,443	0,052	0,008	115	6 - 16–3 - 1	0,426	0,087	0,009	171	6 - 4–17 - 1	0,497	0,299	0,011
4	6 - 20–11 - 1	0,382	0,038	0,007	60	6 - 15–1 - 1	0,439	0,121	0,008	116	6 - 10–10 - 1	0,454	0,104	0,009	172	6 - 6–16 - 1	0,445	0,218	0,011
5	6 - 18–5 - 1	0,391	0,047	0,007	61	6 - 18–3 - 1	0,444	0,043	0,008	117	6 - 11–16 - 1	0,408	0,065	0,009	173	6 - 6–15 - 1	0,495	0,071	0,011
6	6 - 19–11 - 1	0,436	0,041	0,007	62	6 - 17–2 - 1	0,417	0,045	0,008	118	6 - 8–20 - 1	0,420	0,194	0,009	174	6 - 6–7 - 1	0,463	0,172	0,011
7	6 - 19–18 - 1	0,438	0,042	0,007	63	6 - 15–14 - 1	0,420	0,066	0,008	119	6 - 14–3 - 1	0,477	0,041	0,009	175	6 - 5–20 - 1	0,485	0,106	0,011
8	6 - 20–13 - 1	0,433	0,051	0,007	64	6 - 11–4 - 1	0,352	0,053	0,008	120	6 - 9–16 - 1	0,482	0,112	0,009	176	6 - 9–7 - 1	0,402	0,065	0,011
9	6 - 17–9 - 1	0,416	0,060	0,007	65	6 - 14–18 - 1	0,398	0,141	0,008	121	6 - 15–3 - 1	0,400	0,049	0,009	177	6 - 8–15 - 1	0,429	0,121	0,011
10	6 - 19–14 - 1	0,439	0,085	0,007	66	6 - 9–8 - 1	0,482	0,059	0,008	122	6 - 9–2 - 1	0,396	0,065	0,009	178	6 - 8–5 - 1	0,396	0,054	0,012
11	6 - 20–19 - 1	0,412	0,042	0,007	67	6 - 15–2 - 1	0,329	0,045	0,008	123	6 - 11–17 - 1	0,398	0,051	0,009	179	6 - 5–17 - 1	0,429	0,225	0,012
12	6 - 18–19 - 1	0,422	0,063	0,007	68	6 - 20–17 - 1	0,352	0,044	0,008	124	6 - 7–20 - 1	0,435	0,069	0,009	180	6 - 5–8 - 1	0,437	0,312	0,012
13	6 - 17–4 - 1	0,417	0,091	0,007	69	6 - 9–14 - 1	0,490	0,060	0,008	125	6 - 8–18 - 1	0,460	0,187	0,009	181	6 - 5–15 - 1	0,370	0,215	0,012
14	6 - 20–8 - 1	0,392	0,046	0,007	70	6 - 14–9 - 1	0,367	0,049	0,008	126	6 - 10–1 - 1	0,340	0,081	0,009	182	6 - 4–20 - 1	0,491	0,178	0,012
15	6 - 18–7 - 1	0,390	0,079	0,007	71	6 - 12–12 - 1	0,424	0,067	0,008	127	6 - 12–16 - 1	0,438	0,045	0,009	183	6 - 7–3 - 1	0,410	0,147	0,012
16	6 - 14–14 - 1	0,423	0,085	0,007	72	6 - 16–5 - 1	0,388	0,070	0,008	128	6 - 10–17 - 1	0,425	0,138	0,009	184	6 - 4–13 - 1	0,453	0,269	0,012
17	6 - 18–8 - 1	0,363	0,055	0,007	73	6 - 13–5 - 1	0,450	0,071	0,008	129	6 - 7–1 - 1	0,430	0,084	0,009	185	6 - 6–18 - 1	0,490	0,221	0,012
18	6 - 19–4 - 1	0,365	0,045	0,007	74	6 - 8–14 - 1	0,445	0,076	0,008	130	6 - 10–14 - 1	0,414	0,112	0,009	186	6 - 4–4 - 1	0,446	0,483	0,012
19	6 - 20–7 - 1	0,440	0,087	0,007	75	6 - 17–18 - 1	0,432	0,095	0,008	131	6 - 9–15 - 1	0,442	0,149	0,009	187	6 - 3–12 - 1	0,491	0,216	0,012
20	6 - 20–6 - 1	0,451	0,044	0,007	76	6 - 8–8 - 1	0,486	0,254	0,008	132	6 - 16–16 - 1	0,430	0,093	0,009	188	6 - 3–8 - 1	0,465	0,102	0,013
21	6 - 15–13 - 1	0,431	0,087	0,007	77	6 - 13–4 - 1	0,423	0,071	0,008	133	6 - 11–19 - 1	0,467	0,107	0,009	189	6 - 6–3 - 1	0,469	0,066	0,013
22	6 - 18–6 - 1	0,357	0,046	0,007	78	6 - 14–8 - 1	0,439	0,064	0,008	134	6 - 13–16 - 1	0,459	0,084	0,010	190	6 - 12–5 - 1	0,373	0,052	0,013
23	6 - 16–13 - 1	0,467	0,045	0,007	79	6 - 20–10 - 1	0,410	0,043	0,008	135	6 - 8–10 - 1	0,404	0,053	0,010	191	6 - 4–1 - 1	0,486	0,114	0,013
24	6 - 13–17 - 1	0,463	0,069	0,007	80	6 - 12–4 - 1	0,439	0,081	0,008	136	6 - 9–3 - 1	0,474	0,145	0,010	192	6 - 3–20 - 1	0,476	0,353	0,013
25	6 - 17–17 - 1	0,425	0,045	0,008	81	6 - 15–20 - 1	0,444	0,096	0,008	137	6 - 18–1 - 1	0,337	0,083	0,010	193	6 - 6–20 - 1	0,468	0,059	0,013
26	6 - 16–6 - 1	0,413	0,053	0,008	82	6 - 17–20 - 1	0,421	0,058	0,008	138	6 - 6–1 - 1	0,477	0,335	0,010	194	6 - 3–2 - 1	0,474	0,158	0,013
27	6 - 19–9 - 1	0,422	0,174	0,008	83	6 - 13–11 - 1	0,395	0,094	0,008	139	6 - 8–3 - 1	0,390	0,066	0,010	195	6 - 3–6 - 1	0,498	0,180	0]014
28	6 - 13–8 - 1	0,419	0,048	0,008	84	6 - 17–6 - 1	0,428	0,054	0,008	140	6 - 8–16 - 1	0,396	0,127	0,010	196	6 - 20 - 1	0,327	0,066	0,014
29	6 - 13–18 - 1	0,391	0,054	0,008	85	6 - 15–12 - 1	0,432	0,043	0,008	141	6 - 10–12 - 1	0,424	0,124	0,010	197	6 - 4–10 - 1	0,485	0,177	0,014
30	6 - 16–10 - 1	0,383	0,052	0,008	86	6 - 14–2 - 1	0,391	0,132	0,008	142	6 - 10–6 - 1	0,461	0,073	0,010	198	6 - 8–19 - 1	0,452	0,099	0,014
31	6 - 13–20 - 1	0,449	0,112	0,008	87	6 - 12–1 - 1	0,448	0,044	0,008	143	6 - 6–4 - 1	0,418	0,068	0,010	199	6 - 15 - 1	0,323	0,073	0,014
32	6 - 12–11 - 1	0,454	0,116	0,008	88	6 - 17–8 - 1	0,361	0,044	0,008	144	6 - 5–14 - 1	0,478	0,207	0,010	200	6 - 15–15 - 1	0,472	0,045	0,015
33	6 - 17–19 - 1	0,381	0,120	0,008	89	6 - 11–2 - 1	0,467	0,092	0,008	145	6 - 7–17 - 1	0,449	0,206	0,010	201	6 - 19 - 1	0,317	0,105	0,015
34	6 - 19–2 - 1	0,358	0,068	0,008	90	6 - 13–9 - 1	0,445	0,086	0,008	146	6 - 9–11 - 1	0,458	0,090	0,010	202	6 - 4–8 - 1	0,438	0,100	0,015
35	6 - 16–15 - 1	0,393	0,088	0,008	91	6 - 17–13 - 1	0,408	0,045	0,008	147	6 - 8–7 - 1	0,441	0,062	0,010	203	6 - 19–3 - 1	0,412	0,052	0,015
36	6 - 16–17 - 1	0,435	0,092	0,008	92	6 - 10–5 - 1	0,432	0,056	0,008	148	6 - 7–19 - 1	0,432	0,069	0,010	204	6 - 15–10 - 1	0,443	0,090	0,016
37	6 - 19–5 - 1	0,386	0,083	0,008	93	6 - 18–11 - 1	0,456	0,085	0,008	149	6 - 5–4 - 1	0,459	0,151	0,010	205	6 - 11–10 - 1	0,393	0,065	0,016
38	6 - 20–16 - 1	0,357	0,048	0,008	94	6 - 14–12 - 1	0,428	0,067	0,008	150	6 - 10–2 - 1	0,415	0,054	0,010	206	6 - 9–5 - 1	0,477	0,112	0,016
39	6 - 16–7 - 1	0,419	0,056	0,008	95	6 - 17–15 - 1	0,461	0,051	0,008	151	6 - 6–9 - 1	0,454	0,091	0,010	207	6 - 6–17 - 1	0,498	0,254	0,017
40	6 - 19–20 - 1	0,415	0,077	0,008	96	6 - 10–11 - 1	0,433	0,052	0,008	152	6 - 7–13 - 1	0,472	0,063	0,010	208	6 - 11 - 1	0,377	0,154	0,017
41	6 - 18–15 - 1	0,396	0,114	0,008	97	6 - 10–16 - 1	0,469	0,097	0,008	153	6 - 11–9 - 1	0,373	0,053	0,010	209	6 - 14 - 1	0,349	0,083	0,017
42	6 - 17–14 - 1	0,390	0,052	0,008	98	6 - 11–11 - 1	0,414	0,053	0,008	154	6 - 9–1 - 1	0,434	0,055	0,010	210	6 - 18–13 - 1	0,400	0,044	0,018
43	6 - 16–9 - 1	0,386	0,047	0,008	99	6 - 9–10 - 1	0,434	0,112	0,008	155	6 - 11–6 - 1	0,410	0,053	0,010	211	6 - 5 - 1	0,336	0,110	0,019
44	6 - 18–12 - 1	0,419	0,049	0,008	100	6 - 10–20 - 1	0,498	0,056	0,008	156	6 - 7–15 - 1	0,361	0,062	0,011	212	6 - 20–9 - 1	0,421	0,050	0,022
45	6 - 16–19 - 1	0,406	0,077	0,008	101	6 - 12–6 - 1	0,433	0,088	0,008	157	6 - 7–14 - 1	0,482	0,105	0,011	213	6 - 6 - 1	0,409	0,181	0,022
46	6 - 11–18 - 1	0,452	0,086	0,008	102	6 - 10–18 - 1	0,456	0,101	0,009	158	6 - 9–6 - 1	0,473	0,150	0,011	214	6 - 10 - 1	0,387	0,122	0,022
47	6 - 18–18 - 1	0,431	0,128	0,008	103	6 - 12–13 - 1	0,465	0,112	0,009	159	6 - 13–3 - 1	0,394	0,054	0,011	215	6 - 3 - 1	0,454	0,196	0,023
48	6 - 17–12 - 1	0,405	0,045	0,008	104	6 - 13–2 - 1	0,459	0,072	0,009	160	6 - 5–6 - 1	0,468	0,090	0,011	216	6 - 2 - 1	0,360	0,257	0,023
49	6 - 15–16 - 1	0,430	0,056	0,008	105	6 - 16–12 - 1	0,394	0,054	0,009	161	6 - 8–4 - 1	0,448	0,057	0,011	217	6 - 9–4 - 1	0,446	0,098	0,026
50	6 - 16–14 – 1	0,418	0,049	0,008	106	6 - 11–3 - 1	0,475	0,126	0,009	162	6 - 4–5 - 1	0,454	0,065	0,011	218	6 - 15–11 - 1	0,388	0,153	0,027
51	6 - 16–4 – 1	0,446	0,074	0,008	107	6 - 15–7 - 1	0,429	0,094	0,009	163	6 - 5–10 - 1	0,447	0,138	0,011	219	6 - 4 - 1	0,477	0,187	0,027
52	6 - 13–10 - 1	0,424	0,049	0,008	108	6 - 10–19 - 1	0,407	0,109	0,009	164	6 - 7–10 - 1	0,462	0,118	0,011	220	6 - 19–10 - 1	0,408	0,106	0,028
53	6 - 18–20 - 1	0,444	0,049	0,008	109	6 - 11–7 - 1	0,440	0,044	0,009	165	6 - 8–12 - 1	0,443	0,094	0,011	221	6 - 15–19 - 1	0,445	0,094	0,029
54	6 - 15–4 - 1	0,449	0,045	0,008	110	6 - 14–4 - 1	0,436	0,051	0,009	166	6 - 10–7 - 1	0,412	0,110	0,011	222	6 - 15–17 - 1	0,351	0,066	0,029
55	6 - 14–10 - 1	0,364	0,048	0,008	111	6 - 19–13 - 1	0,377	0,091	0,009	167	6 - 6–12 - 1	0,480	0,077	0,011	223	6 - 3–4 - 1	0,479	0,161	0,030
56	6 - 20–3 - 1	0,356	0,047	0,008	112	6 - 11–14 - 1	0,428	0,082	0,009	168	6 - 6–5 - 1	0,408	0,139	0,011	224	6 - 6–13 - 1	0,403	0,272	0,030
**No.**	**ANN architecture**	**MSE 50 epochs**	**MSE 500 epochs**	**MSE 5000 epochs**	**No.**	**ANN architecture**	**MSE 50 epochs**	**MSE 500 epochs**	**MSE 5000 epochs**	**No.**	**ANN architecture**	**MSE 50 epochs**	**MSE 500 epochs**	**MSE 5000 epochs**	**No.**	**ANN architecture**	**MSE 50 epochs**	**MSE 500 epochs**	**MSE 5000 epochs**
225	6 - 15–8 - 1	0,357	0,051	0,031	281	6 - 7–8 - 1	0,465	0,197	0,057	337	6 - 7 - 1	0,421	0,111	0,065	393	6 - 5–3 - 1	0,504	0,165	0,216
226	6 - 10–15 - 1	0,451	0,084	0,033	282	6 - 14–13 - 1	0,398	0,056	0,057	338	6 - 3–9 - 1	0,501	0,262	0,065	394	6 - 3–14 - 1	0,500	0,263	0,218
227	6 - 20–1 - 1	0,369	0,043	0,034	283	6 - 8–2 - 1	0,410	0,117	0,057	339	6 - 3–15 - 1	0,489	0,185	0,065	395	6 - 2–6 - 1	0,497	0,405	0,232
228	6 - 17–1 - 1	0,388	0,064	0,036	284	6 - 17–16 - 1	0,405	0,094	0,057	340	6 - 18–17 - 1	0,441	0,076	0,067	396	6 - 5–9 - 1	0,495	0,107	0,232
229	6 - 15–5 - 1	0,401	0,057	0,036	285	6 - 19–6 - 1	0,450	0,047	0,057	341	6 - 16–2 - 1	0,441	0]052	0,068	397	6 - 2–18 - 1	0,494	0,227	0,250
230	6 - 7–16 - 1	0,404	0,241	0,037	286	6 - 18–4 - 1	0,387	0,053	0,057	342	6 - 9–18 - 1	0,409	0,075	0,070	398	6 - 1–18 - 1	0,501	0,495	0,251
231	6 - 10–4 - 1	0,456	0,114	0,037	287	6 - 12–14 - 1	0,486	0,131	0,057	343	6 - 13–6 - 1	0,478	0,063	0,071	399	6 - 1–16 - 1	0,495	0,494	0,252
232	6 - 16–20 - 1	0,432	0,056	0,037	288	6 - 15–6 - 1	0,471	0,047	0,057	344	6 - 17–7 - 1	0,391	0,089	0,084	400	6 - 1–10 - 1	0,499	0,489	0,254
233	6 - 14–20 - 1	0,389	0,050	0,038	289	6 - 8–13 - 1	0,476	0,071	0,057	345	6 - 20–12 - 1	0,410	0,037	0,085	401	6 - 1–8 - 1	0,487	0,489	0,265
234	6 - 20–20 - 1	0,428	0,076	0,039	290	6 - 12–10 - 1	0,362	0,053	0,057	346	6 - 9 - 1	0,349	0,201	0,086	402	6 - 2–8 - 1	0,462	0,235	0,283
235	6 - 19–1 - 1	0,417	0,132	0,042	291	6 - 14–16 - 1	0,433	0,056	0,057	347	6 - 8–1 - 1	0,434	0,097	0,090	403	6 - 2–13 - 1	0,435	0,270	0,297
236	6 - 6–14 - 1	0,428	0,063	0,042	292	6 - 13–12 - 1	0,448	0,057	0,057	348	6 - 4–7 - 1	0,443	0,211	0,091	404	6 - 2–20 - 1	0,419	0,326	0,298
237	6 - 12–2 - 1	0,399	0,059	0,043	293	6 - 12 - 1	0,342	0,087	0,057	349	6 - 3–5 - 1	0,479	0,247	0,094	405	6 - 1–5 - 1	0,490	0,264	0,326
238	6 - 20–4 - 1	0,446	0,064	0,044	294	6 - 5–16 - 1	0,477	0,062	0,057	350	6 - 16–1 - 1	0,425	0,121	0,096	406	6 - 1–20 - 1	0,497	0,440	0,328
239	6 - 18–14 - 1	0,352	0,038	0,044	295	6 - 13–1 - 1	0,352	0,052	0,058	351	6 - 2–10 - 1	0,399	0,318	0,097	407	6 - 3–7 - 1	0,452	0,227	0,330
240	6 - 18 - 1	0,300	0,141	0,045	296	6 - 5–19 - 1	0,456	0,075	0,058	352	6 - 2–3 - 1	0,497	0,362	0,097	408	6 - 2–17 - 1	0,495	0,347	0,344
241	6 - 14–19 - 1	0,410	0,049	0,045	297	6 - 16–18 - 1	0,411	0,045	0,058	353	6 - 3–16 - 1	0,473	0,207	0,098	409	6 - 1–11 - 1	0,501	0,307	0,371
242	6 - 14–7 - 1	0,432	0,058	0,046	298	6 - 6–8 - 1	0,443	0,109	0,058	354	6 - 4–12 - 1	0,486	0,310	0,098	410	6 - 1–12 - 1	0,495	0,249	0,371
243	6 - 18–9 - 1	0,384	0,045	0,047	299	6 - 12–19 - 1	0,473	0,057	0,058	355	6 - 17–11 - 1	0,363	0,061	0,104	411	6 - 1–2 - 1	0,495	0,435	0,382
244	6 - 9–20 - 1	0,377	0,050	0,048	300	6 - 7–7 - 1	0,463	0,126	0,058	356	6 - 17–3 - 1	0,388	0,048	0,106	412	6 - 1–13 - 1	0,494	0,348	0,393
245	6 - 10–3 - 1	0,462	0,110	0,048	301	6 - 8–11 - 1	0,410	0,102	0,058	357	6 - 14–1 - 1	0,415	0,049	0,107	413	6 - 1 - 1	0,435	0,486	0,408
246	6 - 10–9 - 1	0,502	0,052	0,048	302	6 - 12–17 - 1	0,421	0,048	0,058	358	6 - 18–2 - 1	0,353	0,079	0,107	414	6 - 1–1 - 1	0,511	0,284	0,465
247	6 - 20–18 - 1	0,447	0,048	0,049	303	6 - 9–12 - 1	0,391	0,116	0,059	359	6 - 7–18 - 1	0,483	0,101	0,107	415	6 - 1–6 - 1	0,488	0,301	0,476
248	6 - 12–8 - 1	0,413	0,048	0,049	304	6 - 11–12 - 1	0,473	0,083	0,059	360	6 - 15–18 - 1	0,443	0,065	0,107	416	6 - 1–15 - 1	0,489	0,464	0,479
249	6 - 8–17 - 1	0,478	0,115	0,050	305	6 - 13–19 - 1	0,390	0,064	0,059	361	6 - 3–3 - 1	0,486	0,495	0,109	417	6 - 1–3 - 1	0,478	0,381	0,488
250	6 - 7–2 - 1	0,470	0,062	0,050	306	6 - 10–13 - 1	0,439	0,080	0,059	362	6 - 9–13 - 1	0,439	0,090	0,110	418	6 - 1–4 - 1	0,494	0,486	0,495
251	6 - 14–6 - 1	0,441	0,048	0,050	307	6 - 5–12 - 1	0,477	0,279	0,059	363	6 - 2–12 - 1	0,499	0,356	0,110	419	6 - 1–7 - 1	0,484	0,427	0,496
252	6 - 20–14 - 1	0,403	0,043	0,051	308	6 - 10–8 - 1	0,460	0,056	0,059	364	6 - 5–7 - 1	0,428	0,163	0,110	420	6 - 1–19 - 1	0,500	0,495	0,500
253	6 - 13–14 - 1	0,427	0,081	0,051	309	6 - 9–9 - 1	0,417	0,075	0,059	365	6 - 4–15 - 1	0,459	0,291	0,112	421	6 - 1–14 - 1	0,500	0,495	0,501
254	6 - 12–20 - 1	0,440	0,050	0,051	310	6 - 3–11 - 1	0,468	0,138	0,059	366	6 - 2–15 - 1	0,468	0,334	0,113					
255	6 - 14–5 - 1	0,403	0,045	0,052	311	6 - 7–5 - 1	0,455	0,071	0,060	367	6 - 6–10 - 1	0,445	0,070	0,114					
256	6 - 8–6 - 1	0,449	0,053	0,052	312	6 - 6–2 - 1	0,496	0,087	0,060	368	6 - 5–13 - 1	0,418	0,353	0,114					
257	6 - 14–11 - 1	0,422	0,072	0,053	313	6 - 12–9 - 1	0,406	0,125	0,060	369	6 - 5–18 - 1	0,449	0,331	0,115					
258	6 - 13–15 - 1	0,430	0,071	0,053	314	6 - 4–6 - 1	0,406	0,269	0,060	370	6 - 4–19 - 1	0,482	0,183	0,116					
259	6 - 7–9 - 1	0,453	0,106	0,053	315	6 - 5–5 - 1	0,482	0,068	0,060	371	6 - 5–2 - 1	0,476	0,216	0,118					
260	6 - 17–10 - 1	0,464	0,047	0,053	316	6 - 6–11 - 1	0,487	0,075	0,060	372	6 - 2–14 - 1	0,480	0,279	0,119					
261	6 - 12–18 - 1	0,439	0,051	0,053	317	6 - 6–19 - 1	0,361	0]121	0,061	373	6 - 20–15 - 1	0,414	0,048	0,128					
262	6 - 11–15 - 1	0,367	0,096	0,053	318	6 - 4–2 - 1	0,465	0,190	0,061	374	6 - 4–14 - 1	0,439	0,074	0,130					
263	6 - 17–5 - 1	0,464	0,132	0,053	319	6 - 5–1 - 1	0,410	0,131	0,061	375	6 - 3–13 - 1	0,492	0,277	0,134					
264	6 - 11–13 - 1	0,491	0,154	0,054	320	6 - 7–6 - 1	0,496	0,113	0,061	376	6 - 4–18 - 1	0,462	0,136	0,135					
265	6 - 7–11 - 1	0,458	0,062	0,054	321	6 - 9–17 - 1	0,432	0,146	0,061	377	6 - 2–9 - 1	0,469	0,433	0,136					
266	6 - 19–16 - 1	0,424	0,165	0,055	322	6 - 3–19 - 1	0,488	0,285	0,061	378	6 - 1–17 - 1	0,456	0,382	0,141					
267	6 - 16 - 1	0,327	0,064	0,055	323	6 - 2–19 - 1	0,486	0,279	0,061	379	6 - 4–11 - 1	0,389	0,150	0,141					
268	6 - 20–5 - 1	0,427	0,076	0,055	324	6 - 3–10 - 1	0,495	0,250	0,061	380	6 - 1	0,300	0,218	0,143					
269	6 - 11–8 - 1	0,456	0,054	0,055	325	6 - 4–9 - 1	0,436	0,222	0,062	381	6 - 3–18 - 1	0,392	0,228	0,143					
270	6 - 14–15 - 1	0,422	0,060	0,055	326	6 - 9–19 - 1	0,464	0,098	0,062	382	6 - 4–16 - 1	0,461	0,081	0,143					
271	6 - 13–7 - 1	0,439	0,049	0,055	327	6 - 7–12 - 1	0,418	0,219	0,062	383	6 - 2–2 - 1	0,450	0,451	0,151					
272	6 - 17 - 1	0,350	0,079	0,055	328	6 - 2–1 - 1	0,503	0,495	0,062	384	6 - 2–5 - 1	0,488	0,354	0,152					
273	6 - 12–15 - 1	0,470	0,048	0,055	329	6 - 8 - 1	0,411	0,119	0,062	385	6 - 11–5 - 1	0,483	0,099	0,154					
274	6 - 19–7 - 1	0,420	0,049	0,056	330	6 - 4–3 - 1	0,504	0,288	0,062	386	6 - 2–7 - 1	0,495	0,365	0,168					
275	6 - 15–9 - 1	0,474	0,058	0,056	331	6 - 14–17 - 1	0,377	0,092	0,063	387	6 - 3–17 - 1	0,395	0,306	0,170					
276	6 - 11–20 - 1	0,369	0,063	0,056	332	6 - 12–3 - 1	0,451	0,063	0,063	388	6 - 3–1 - 1	0,476	0,203	0,177					
277	6 - 19–12 - 1	0,414	0,043	0,056	333	6 - 13 - 1	0,298	0,128	0,063	389	6 - 2–11 - 1	0,493	0,319	0,181					
278	6 - 19–17 - 1	0,404	0,093	0,056	334	6 - 13–13 - 1	0,479	0,048	0,064	390	6 - 1–9 - 1	0,495	0,485	0,209					
279	6 - 20–2 - 1	0,425	0,080	0,056	335	6 - 7–4 - 1	0,453	0,134	0,064	391	6 - 2–4 - 1	0,496	0,426	0,212					
280	6 - 19–8 - 1	0,420	0,065	0,056	336	6 - 2–16 - 1	0,485	0,284	0,064	392	6 - 6–6 - 1	0,442	0,194	0,214				,	

The models with the least MSE are marked in bold.

## Results

The data have been analyzed using NeuronDotNet computer library [32]. Training an ANN is the process of setting the best weights on the inputs of each of the nodes. The goal is to use the training set to produce weights where the output of the network is as close to the desired output as possible for as many of the examples in the training set as possible [30]. Table 3 shows the MSE for all 421 trained artificial neural models. A satisfactory MSE was yielded for ANNs with:

18 nodes in the first hidden layer and 16 nodes in the second hidden layer

19 nodes in the first hidden layer and 19 nodes in the second hidden layer

18 nodes in the first hidden layer and 10 nodes in the second hidden layer

**Figure 2:**
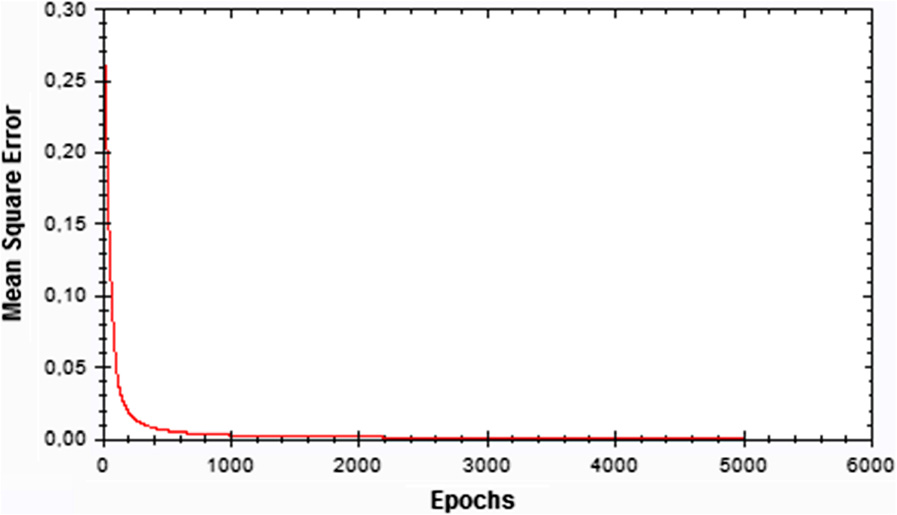
**Plot of total error in training ANN based on 6**-**18**-**16**-**1 architecture.** Training of the feedforward backpropagation neural network as measured by the square error of the difference between the actual and predicted variable.

Figure 2 presents the MSE for ANN model based on 6-18-16-1 architecture and the training set.

Table 2 lists classification results on the test set of ANN modelling for presence and absence of Idiopathic Scoliosis in the anamnesis. It proves how well the artificial neural network will perform on new data. The comparison of developed models showed, that the most satisfactory classification accuracy was achieved for ANN model with 18 nodes in the first hidden layer and 16 nodes in the second hidden layer. The classification accuracy for Idiopathic Scoliosis in the anamnesis with expression measurement of ACTB and GAPDH with use of ANN based on 6-18-16-1 architecture was 8 of 9 (88%). Only in one case (ID 27 in test set), the prediction was ambiguous.

## Conclusions

The results of this study confirm the potential benefits of the artificial neural network application for clinical research and point at human housekeeping genes as a potential target for future molecular investigations on idiopathic scoliosis etiopathogenesis. The analysis indicates the relationship between level of expression of ACTB, GAPDH and familial Idiopathic Scoliosis.

## Abbreviations

IS: Idiopathic Scoliosis;ANN: Artificial neural network;mRNA: Messenger ribonucleic acid;QRT PCR: Quantitative Real Time Reverse Transciptase Chain Reaction

## Competing interests

The authors declare that they have no financial or non-financial competing interests.

## Authors' contributions

RN participated in the design of the study, performed spinal surgeries, prepared tissue samples, performed radiological measurements and statistical analysis and drafted the manuscript. TW carried out analysis based on artificial neural network and participated in the design of the study. MT has supported us with her experience and knowledge concerning advanced data analysis: knowledge discovery from data, data mining, artificial intelligence and machine learning, and together with DZ and UM has been involved in the design of the study and interpretation of the data and helped to draft the manuscript. All authors read and approved the final manuscript.
